# Application of encapsulated *Indigofera tinctoria* extract as a natural antioxidant and colorant in ice cream

**DOI:** 10.1002/fsn3.3228

**Published:** 2023-01-11

**Authors:** Talieh Shadordizadeh, Elham Mahdian, Mohammad Ali Hesarinejad

**Affiliations:** ^1^ Department of Food Science and Technology, Quchan Branch Islamic Azad University Quchan Iran; ^2^ Department of Food Processing Research Institute of Food Science and Technology (RIFST) Mashhad Iran

**Keywords:** antioxidant, color, encapsulation, ice cream, *Indigofera tinctoria*

## Abstract

In this study, *Indigofera tinctoria* extract (ITLE) along with maltodextrin in different concentrations was encapsulated using a freeze dryer, and some physicochemical properties were measured. Powder containing 30% maltodextrin was selected as the optimal powder for use in ice‐cream production in four levels (0%–1.8%), and some quality parameters were examined. The results showed that with increasing the carrier concentration, moisture content, a_w_, solubility, a*, and b* of the powders decreased; bulk density, tapped density, and L* did not change significantly; total phenolic content and antioxidant activity of powders also increased significantly (*p* < .05). Addition of encapsulated ITLE to the ice cream caused a significant decrease in L*, b*, and melting rate, a significant increase in a*, overrun, and hardness of the samples and no change in the viscosity of the ice‐cream mix (*p* < .05). Ice cream containing 1.2% encapsulated ITLE had higher sensory acceptance than other levels following control ice cream. The results of this study showed that ITLE can be used as a desirable additive in the production as a natural antioxidant and color agent.

## Graphical abstract

In this study, Indigofera tinctoria extract along with maltodextrin in different concentrations was encapsulated using a freeze dryer, and some physicochemical properties were measured. Final powder was used in ice‐cream production, and some quality parameters were examined. The results of this study showed that Indigofera tinctoria extract encapsulated can be used as a desirable additive in the production as a natural antioxidant and color agent.

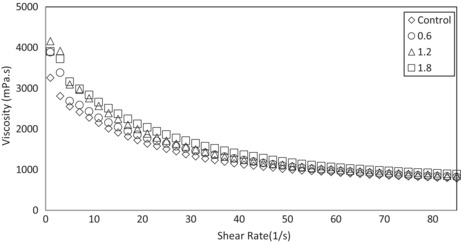

## 1 INTRODUCTION

One of the most crucial preference criteria for assessing consumer acceptability of a product is its appearance. The first impression of food products is their appearance; therefore, whether they are rejected or accepted is largely determined by their appearance (Amiryousefi & Hesarinejad, 2021; Arici et al., 2016; Rezaiyan Attar et al., 2021). Among these, color is one of the most essential aspects of foods since it is used as a quality indication to assess whether or not they would be accepted (Azeredo, 2009; Chranioti et al., 2015; Salehi et al., 2021). Due to the importance of color in determining a product's consumer acceptance, manufacturers try to improve the color of the related material by adding natural or synthetic colorants in accordance with regulations. Synthetic colorants have been widely used to color food products; however, their usage in the food industry is a contentious issue due to their toxicological risk to human health (Mizutani, 2009). As a result, due to the hazardous effects of some synthetic colorants, producers prefer to use natural ones instead (Bayat Tork et al., 2022; Chou et al., 2007). Natural colorants may be expected to improve not just product visual features, but also various bioactive (e.g., antioxidant activity) or technical (e.g., preservative) properties; in other words, they may be a “multi‐effect” component (Genc Polat et al., 2020).

Herbs are natural sources of flavor and color, and they have been employed for medicinal purposes for thousands of years. They are also one of the best sources of natural antioxidants because they contain potent compounds that have been demonstrated to bring color to foods while also having an antioxidative effect (Embuscado, 2015; Rezaei et al., 2019). It has also been shown that a wide range of phenolic substances derived from plants and spices have strong antioxidant, anti‐inflammatory, antimutational, anticancer, and antitumor activity (Saini et al., 2013).

Encapsulation is a process of trapping active compounds inside particles and protecting them from environmental factors such as heat, light, or oxidation (Zuidam & Nedovic, 2010). Stabilizing nutrient compounds or increasing their bioavailability is one of the most important reasons for encapsulation of active compounds (Desai & Jin Park, 2005). In other words, in the food industry, encapsulating is a good way to improve the transport of bioactive molecules (antioxidants, minerals, vitamins, phytosterols, lutein, fatty acids, and lycopene) and living cells (probiotics) into foods (de Vos et al., 2010; Hesarinejad, Abdollahi Moghaddam, et al., 2021). Many food ingredients have been finely encapsulated with various technologies, including acidifiers, flavoring agents, sweeteners, colorants, lipids, vitamins, minerals, enzymes, and microorganisms (Desai & Jin Park, 2005).

Ice cream is a significant dietary supplement that affects human health. A more nourishing and healthy ice cream can be produced by adding natural ingredients such as antioxidants. The ice‐cream mix consists mainly of a mixture of milk, emulsifiers, stabilizers, sweeteners, flavorings, and coloring agents (Trgo et al., 1999). Natural food colorants with health benefits have gained popularity in recent years due to their ability to improve the functional and nutritional characteristics of foods (Durmaz et al., 2020). As a result of customer views and preferences, many researchers have worked to investigate natural coloring agents in ice cream (Durmaz et al., 2020; Öztürk et al., 2018; Van Kleef et al., 2002).

*Indigofera tinctoria* L., named “Vasmeh” in Iran, is a member of the Fabaceae family. Since time immemorial, this erect, pubescent shrub has been used in Indian and Chinese medicine to treat a variety of diseases, including cancer, liver ailments, epilepsy, inflammation, ulcers, bronchitis, neurological problems, as ointment for sores, old ulcers, and hemorrhoids (Anusuya & Manian, 2013). Therapeutic properties are generally associated with antioxidant properties. The active ingredient of this plant's leaves, indigrubin, is a potential anticancer medicine (Han, 1994). The furano‐flavonoids (pseudosemiglabrin, semiglabrin, and glabritephrin) and kaempferol‐4′7‐dirhamnoside, a rare flavonoid glycoside derived from this plant, were found to be effective antidyslipidemic drugs (Narender et al., 2006). The natural blue dye (Neel or indigo) derived from Vasmeh's leaflets and branches is well‐known.

Due to the importance of using natural dyes and antioxidant compounds in food and the unique properties of *Indigofera tinctoria* L., the purpose of this study was to use the encapsulated Vasmeh extract in ice cream as a natural dye and antioxidant.

## 2 MATERIALS AND METHODS

### 2.1 Materials

*Indigofera tinctoria* L. leaves were purchased locally from the medical plant market, Mashhad, Iran. Reagent 2‐diphenyl‐1–1‐picrylhydrazyl (DPPH) and Folin–Ciocalteu reagent were prepared from Sigma. Gallic acid was prepared by Merck Company. Maltodextrin (DE of 18–20) was prepared by Foodchem Company (China). Sugar, vanilla, mono‐ and diglyceride, and salep were purchased from a local supplier in Mashhad, Iran. Cream (30% milk fat) and skim milk powder (96% milk solids) were bought from Kalleh Co. (Amol, Iran), Golshad Co. (Mashhad, Iran), respectively.

### 2.2 Preparation of *Indigofera tinctoria* L. extract (ITLE)

After cleaning manually the leaflets and branches to remove all foreign matter such as dust, dirt, stones, and chaff; to achieve uniform particle sizes, the samples were ground and passed through a 0.3 mm mesh sieve. The extraction process was performed by adding 20 g of plant powder in 200 ml of distilled water at room temperature using a magnetic stirrer at 100 rpm overnight. The mixture was filtered through Whatman #4 (Rezaei et al., 2019).

### 2.3 Encapsulation of ITLE

Maltodextrin concentrations of 10%, 20%, and 30% were used to make the freeze‐drying solution, which was labeled M10, M20, and M30, respectively. On a stirrer, ITLE was blended with maltodextrin powder. Before freeze‐drying, the solutions were placed in a 0.75 cm thick layer on petri dishes and frozen for 48 h at 25°C. In a freeze dryer (Operon‐Korea), the frozen samples were dried under vacuum for 48 h at −50°C condenser and 20 mbar absolute pressure. After that, the samples were milled three times in a 10 s on 10 s off mode on a grinder.

### 2.4 Encapsulated powder tests

#### 2.4.1 Moisture content and water activity

The moisture content (MC) of the samples was determined by drying them in an oven (UF55 MEMMERT) at 70°C until they reached a constant weight, as per the AOAC standard (AOAC, 1990). A water activity meter (LabMaster aw, Novasina, Lachen, Switzerland) was used to determine the amount of water activity (a_w_) of samples.

#### 2.4.2 Solubility of powders

The sample (1 g) was transfered to 100 ml of distilled water and agitated at 600 rpm for 5 min on a magnetic stirrer. After that, the dispersion was centrifuged for 10 min at 4000× *g*. At 105°C, the supernatant was dried until it achieved a consistent weight. The difference between the weights of the primary and secondary dry materials was used to compute the powder's solubility percentage (Caparino et al., 2012).

#### 2.4.3 Bulk and tapped densities

Two grams of sample was carefully put into a measuring cylinder and lightly shook to smooth the powder's surface. Finally, the bulk density was calculated using the following equation:

(1) ρ=m/V,

where *m* is the weight of the powder (g) and *V* denotes the volume of the sample (ml).

After measuring the bulk density, the powder volume variations in the cylinder were repeatedly tapped to determine the tapped density. Finally, the tapped density was estimated using the formula *m*/*V* (g/ml) (Naji‐Tabasi et al., 2021).

#### 2.4.4 Color parameters

A colorimeter (Konica Minolta, CR‐410, Japan) was used to determine the color of the powder samples. On the basis of our previous studies, we captured images and processed samples (Hesarinejad, Lorenzo, et al., 2021; Rezagholi & Hesarinejad, 2017). The photographs were taken in a wooden black box using a color digital camera (Canon EOS 1000D, Taiwan) with a resolution of 2272 × 1704 pixels. Image J (National Institutes Health) was used to process the photographs. The RGB images were converted into L*a*b* units, where L*, a*, and b* correspond to lightness (from black to white (0 to 100)), red‐green index (from +60 to −60), and yellow‐blue index (from +60 to −60), respectively.

#### 2.4.5 Determination of antioxidant activity and total phenolic content

The activity of the radical scavenging DPPH was used to determine the antioxidant activity of the samples (Koocheki et al., 2022). The Folin–Ciocalteu technique was used to determine the total phenolic content. The calibration curve was created using the absorbance of gallic acid at 750 nm as the standard compound (Alizadeh Behbahani et al., 2017).

### 2.5 Ice‐cream preparation

Ice‐cream mixes were formulated to contain 12% milk fat, 13% milk solids nonfat (MSNF), 17% sugar, 0.1% vanilla, 0.4% salep, 0.15% mono‐ and diglyceride (MDG), and 0, 0.6, 1.2, and 1.8% ITLE encapsulated powder (control, 0.6ITLE, 1.2ITLE, and 1.8ITLE samples, respectively). The following steps were used to make the ice‐cream samples: First, a domestic mixer (Pars Khazar, Iran) was used to mix cream and skim milk powder (as providers of milk fat and MSNF) with sugar for 2 min. Other ingredients (water, ITLE, MDG, salep, and vanilla) were blended together for 5 min in the second step. The mixtures from Steps 1 and 2 were then blended together for 1 min to generate a homogenous system, pasteurized at 80°C for 25 s (HTST), homogenized at 18,000 rpm for 2 min (Ultra Turrax T25D IKA, Germany), cooled rapidly to 4°C, and aged at 4°C for 6 h. The ice‐cream mixtures were then frozen for 15 ± 2 min in a batch ice‐cream machine (Model ICK5000; Delonghi, Italy) and hardened for around 24 h in a freezer (−18°C) (Ghaderi et al., 2021).

### 2.6 Ice‐cream properties

#### 2.6.1 Rheological measurements

A rotational viscometer (model RVDV‐II; Brookfield Engineering Inc., USA) was used to run rheological tests on the ice‐cream mixtures at a temperature of 25 ± 0.1°C. Prior to measurement, the mixtures were sheared for 800 s at 150 s^−1^ to eliminate the time dependency. The flow behavior data were then obtained by increasing the shear rate (1 to 85 s^−1^) and analyzed using the Herschel–Bulkley model:

(2) $$\tau =\tau_{0}+k{\dot{\gamma }}^{n},$$

where *τ*_0_ is the Herschel–Bulkley yield stress (Pa), k stands for the Herschel–Bulkley consistency coefficient (Pa s^n^), and n shows the Herschel–Bulkley flow behavior index (dimensionless).

#### 2.6.2 Overrun

The overrun was computed as follows:

(3) $$\text{Overrun}\left(\%\right)=\left(\frac{\text{mass of the unit volume of the}\;\mathrm{mix}-\text{mass of the unit volume of the}\;\mathrm{ice}\;\text{cream}\;}{\text{mass of the unit volume of the}\;\mathrm{ice}\;\text{cream}\;}\right)\times 100.$$

#### 2.6.3 Melting rate determination

Thirty grams of ice‐cream samples was placed on a wire rack to melt at 25 ± 1°C. During 60 min, at 5‐min intervals, the weight of molten material was measured. The slope of the linear section of the drained mass‐time graphs was used to determine the melting rate (g/min).

#### 2.6.4 Textural analysis

A texture analyzer (CT3 Texture Analyzer; Brookfield, The USA) fitted with a conical probe (45°) at room temperature was used to perform penetration test on the ice creams. The penetration depth and speed were considered 15 mm and 2 mm/s, respectively. Hardness was measured as the peak compression force (N) during penetration, and adhesiveness was evaluated as the negative surface area (N s) following withdrawal.

#### 2.6.5 Sensory evaluation

The ice creams (−18°C, 30 g) were coded with three‐digit random numbers and presented to 10 panelists (5 females and 5 males between the ages of 25 and 36) over the course of two sessions. Quantitative descriptive analysis (QDA) was used to identify the sensory characteristics of the ice‐cream samples (Stone & Sidel, 2004) (Table 1). A 10‐cm line scale with the words “lowest” and “highest” at its end points was used to rate the intensity of each characteristic.

**TABLE 1:** Sensory attributes and definitions for the evaluation of ice‐cream samples

Attributes	Definitions
Color	Light blue to dark blue under white light
Coarseness	A rough sensation in mouth due to the presence of detectable ice crystals which disappears as the ice crystals melt
Coldness	The chilling of tongue and palate soon after the sample is placed in mouth
Hardness	The resistance against scooping a portion of ice cream

### 2.7 Statistical analysis

The data were analyzed by one‐way analysis of variance (ANOVA, *p* < .05) using the Minitab statistical software (version 18; Minitab Inc.).

## 3 RESULTS AND DISCUSSION

### 3.1 Encapsulated ITLE tests

#### 3.1.1 Moisture content

The effect of maltodextrin concentration on the MC of encapsulated extract powder was significant (*p* < .05). So that with increasing the concentration of maltodextrin, the MC of the samples decreased. The MC of Vasmeh extract powder was in the range of 1.21% to 3.14% (Table 2), in which the oxidative degradation and microbial activity are greatly reduced. Less moisture in the materials reduces adhesion. The highest MC (3.14%) was in the control sample, and the lowest (1.21%) was in the M30.

**TABLE 2:** Characterization of encapsulated *Indigofera tinctoria* L. extract by freeze‐drying method

Encapsules	MC (%)	a_w_ (−)	Solubility (%)	Bulk density (g/ml)	Tapped density (g/ml)	DPPH (mg/100 g)	TPC (mgGAL/100 g)	Color parameters		
								L*	a*	b*
Control	3.14^a^	0.185^b^	96.7^a^	0.61^a^	0.64^a^	60.42^d^	1049.27^d^	31.16^b^	1.39^b^	2.54^c^
M10	2.69^b^	0.241^a^	96.4^b^	0.59^a^	0.60^b^	62.25^c^	1305.64^c^	47.08^a^	2.71^a^	4.61^b^
M20	1.74^c^	0.182^b^	96.3^b^	0.55^a^	0.58^b^	70.37^b^	1985.05^b^	50.85^a^	2.92^a^	6.10^a^
M30	1.21^d^	0.139^c^	95.9^c^	0.54^a^	0.58^b^	75.60^a^	3606.75^a^	55.28^a^	3.02^a^	6.39^a^

^a–d^ Means of two replicates in the same row with same superscripts do not differ significantly (*p* > .05).

In the encapsulating process, the MC of the feed affects the final MC of the product. Adding maltodextrin to the feed before drying increases the total solid content and reduces the amount of free water for evaporation. Therefore, it leads to a decrease in the MC of the powders produced (Grabowski et al., 2006; Mishra et al., 2014; Tuyen et al., 2010). This means that powders with less moisture can be produced by increasing the percentage of maltodextrin added. If the maltodextrin is too high, the product will be of lower quality because the nutrients will be diluted (Quek et al., 2007). According to Fang and Bhandari, moisture acts as a material softener and increasing it will reduce the glass transition temperature (Fang & Bhandari, 2010). The glass transition temperature of dried powders is a good indicator for the stability of the powder in the long‐term storage period. Amorphous solid powder, when present at a storage temperature higher than the glass transition temperature and in the rubber state, has molecular motions and rapid reactions that increase as a result of degradation reactions (Tonon et al., 2010). Therefore, the MC of encapsules has an important role in determining the flow, adhesion, and stability of powders due to the effect of glass transition temperature and crystallization behavior.

Hesarinejad et al. in the study of physicochemical and antioxidant properties of encapsulated *Portulaca oleracea* by spray‐drying method stated that the effect of maltodextrin concentration on the MC of powders was significant. Increasing the concentration of maltodextrin caused a decrease in MC (Hesarinejad, Abdollahi Moghaddam, et al., 2021). The results of the present study were consistent with the studies of Nikjoo et al., and Sarabandi and Peighambardoust on the effect of increasing the concentration of maltodextrin on the moisture content of peppermint and malt extract powder, respectively (Nikjoo et al., 2019; Sarabandi & Peighambardoust, 2015). The reason for this phenomenon can be related to the increase in solids content, the tendency of maltodextrin to absorb moisture, and the decrease in free moisture available for evaporation (Malekizadeh et al., 2018). Gonga et al. also reported that the concentration of wall materials has an important effect on MC (Gong et al., 2007).

#### 3.1.2 Water activity

a_w_ is the ratio of the water vapor pressure of the food system to the pure water vapor pressure at the same temperature (Fennema, 1996). a_w_ is different from moisture; in other words, a_w_ is a measure of the amount of free water available in the food system that is responsible for any biochemical reactions, while the MC indicates the composition of water in the food system. Since the a_w_ of powders affects their shelf life, so the a_w_ is an important indicator for dried powders. In general, foods with an a_w_ of <0.6 are microbiologically stable, and if spoilage occurs, it is the result of chemical reactions, not microorganisms. However, storage conditions also play an important role. The results of a_w_ of encapsulated ITLE showed that increasing the maltodextrin concentration caused a significant decrease in a_w_ (*p* < .05) (Table 2). The highest amount of a_w_ (0.241) was in the M10 sample, and the lowest one (0.139) was in M30. As it was observed, the a_w_ of encapsulated ITLE ranged from 0.139 to 0.241, which indicates the microbiological stability (a_w_ <0.6) of the dried powders and its long shelf life in suitable packaging and storage conditions. According to the results, with increasing the maltodextrin concentration, the a_w_ of the samples decreased (Table 2). As the concentration of the carrier increased, the MC of the powders decreased, thus reducing the amount of free water and, consequently, the a_w_ of the samples. The reason for this decrease could also be attributed to the fact that maltodextrin is linked to water, which had led to a decrease in free water in the system (Hesarinejad, Abdollahi Moghaddam, et al., 2021). These results were consistent with the report of Jangam and Thorat in optimizing ginger powder encapsulation (Jangam & Thorat, 2010).

#### 3.1.3 Solubility

The solubility of the powder is one of the main ways of regenerating and absorbing water. In most powdered foods, the goal is to dissolve the powder quickly and completely in water without the formation of lumps, immersion, and dispersion of particles (Naji‐Tabasi et al., 2021). The results of this study showed that the effect of maltodextrin concentration on the solubility of encapsulated ITLE was significant (*p* < .05). As the concentration of maltodextrin increased, the solubility of the powders decreased (Table 2). These results were in agreement with the results of Mishra et al for amla juice powder (Mishra et al., 2014). However, in contradiction with the results of our study, Cano‐Chauca et al. did not observe a significant difference between the solubility of powder encapsulated with maltodextrin and gum arabic in mango powder (Cano‐Chauca et al., 2005). Some researchers have also suggested that solubility increases with increasing concentration of carriers. This was consistent with findings of Nadeem et al. on the mountain tea powder (Nadeem et al., 2011), Fazaeli et al. on black mulberry juice powder (Fazaeli et al., 2012), and Grabowski et al. on sweetpotato powder (Grabowski et al., 2008).

The results also showed that there is a positive relationship between solubility and MC of powders (*r* = .94). This finding is similar to that of Goula et al. who reported on tomato powder (Goula et al., 2004).

#### 3.1.4 Bulk and tapped densities

The bulk density of particulate solids is a measure of their compaction qualities and can reflect the amount of empty space between powder particles; hence, it is influenced by particle density and arrangement (Sharifi & Poorakbar, 2015). Tapped density is also a very important factor and indicator related to the characteristics of packaging, transportation, and determining the amount of material needed to fill a certain volume of the package (Hesarinejad, Abdollahi Moghaddam, et al., 2021). In industry, bulk and tapped density measurements are used to control storage, processing, packing, and delivery conditions (Hesarinejad, Abdollahi Moghaddam, et al., 2021). Fine particle‐containing masses have much higher bulk and tapped density, as well as lower porosity, than those lacking fine particles. This effect is most likely caused by small particles occupying the pores between the bigger particles. It can also be said that this phenomenon may be due to the properties of maltodextrin, which reduce the adhesion between particles (Zendeboodi et al., 2018). Based on the results of this study, the effect of maltodextrin concentration on bulk density and tapped density was not significant (*p* < .05) (Table 2). With increasing maltodextrin concentration, bulk density and tapped density decreased. In fact, reducing the MC leads to lightening of the particles and thus reducing the bulk density. Abdullah and Geldart stated that as the particle size increases, the adhesion between the particles decreases, and particles with higher flow rate are produced (Abdullah & Geldart, 1999). Particles with high flowability should have a higher bulk density because the interparticle forces become weaker and the powder is packed in a denser condition (Abdullah & Geldart, 1999). Therefore, as mentioned, increasing the concentration of the carrier due to increasing the particle size and also reducing the MC according to the reasons explained reduced the bulk density. On the contrary, due to the fact that the carrier causes the formation of crust on the surface of the particles, increasing the carrier concentration increased the amount of trapped air in the powder structure. Therefore, because water has a higher density than air, samples with lower MC had lower densities (Goula & Adamopoulos, 2010; Rad et al., 2016; Santhalakshmy et al., 2015).

Particle size is an effective factor in tapped density; if the particle size is larger, the volume does not change much due to impact, so the tapped density becomes smaller. Because small particles can be placed in empty spaces (pores), smaller particles will have less volume and higher impact density (Abdullah & Geldart, 1999). Nadeem et al. reported that increasing the concentration of carriers resulted in a 13% increase in bulk density (Nadeem et al., 2011). Mosen et al. attributed the increase in bulk density to the increase in the total solid content of the feed solution (Mosén et al., 2005). By contrast, some studies did not show significant changes with increasing carrier concentrations (Tewa‐Tagne et al., 2007). Also in the study of Mishra et al., the concentration of maltodextrin had no significant effect on the density of Amla powder (Mishra et al., 2014).

#### 3.1.5 Color parameters

The color of the encapsulated powder is an important quality index because it reflects the attractiveness and quality of the powder produced during the encapsulating and drying process and can play an important role in accepting the product in which the powder will be used. Table 2 showed the color parameters of encapsulated ITLE. Based on the results, the addition of maltodextrin increased the lightness of the samples (*p* < .05). The encapsulating process produces porous particles that due to the reduction in density and particle compaction, a bright and shiny color is observed in the powders, which with the increase in carrier concentration, the color of the powders tends to white maltodextrin. Increasing the lightness with increasing the maltodextrin concentration is also a result of the white color of maltodextrin, which with increasing the concentration of maltodextrin, the color of the samples tended to white maltodextrin. The results also showed that with increasing the maltodextrin concentration, the value of a* and b* increased significantly (*p* < .05) (Table 2). Some researchers reported similar results and stated that increasing the amount of maltodextrin increased the L* of pomegranate juice powder (Shahidi et al., 2014), Gac fruit aril powder (Tuyen et al., 2010), watermelon powder (Quek et al., 2007), and *Portulaca oleracea* extract powder (Hesarinejad, Abdollahi Moghaddam, et al., 2021). Abadio et al. also stated that the light yellow color of pineapple juice powders did not change up to a maximum concentration of 15% maltodextrin (Abadio et al., 2004). Quek et al. also reported that an increase of more than 10% of maltodextrin in watermelon juice powder causes a purple color and distances from the desired red color (Quek et al., 2007).

#### 3.1.6 Total phenolic content

The results showed that with increasing the concentration of maltodextrin, the TPC in the powders increased. Phenolic compounds are sensitive to high drying temperatures and therefore lowering the temperature in freeze‐drying prevents thermal decomposition or oxidation and consequently preserves the TPC. It has also been reported that dried powders at low temperatures tend to agglomerate due to their higher MC. This is especially true for powders that are highly sticky in nature due to their high sugar content. Agglomeration results in less exposure of powders to oxygen and thus protects phenolic compounds from decomposition (Horuz et al., 2012; Quek et al., 2007). Some researchers also reported that the use of maltodextrin as a carrier protects phenolic compounds against environmental conditions (Hesarinejad, Abdollahi Moghaddam, et al., 2021; Tuyen et al., 2010). By contrast, Malekizadeh et al. (2018) reported that with increasing the concentration of maltodextrin, the TPC in the encapsulated sumac extract powder decreased. They stated that increasing the concentration of the carrier dilutes the feed entering the dryer such as phenolic compounds in the extract, because maltodextrin is a substance that lacks antioxidant activity, thus reducing the TPC (Malekizadeh et al., 2018).

#### 3.1.7 Antioxidant activity

Free radical scavenging activity is an important feature due to the inhibition of free radicals in food and biological systems and indicates its antioxidant capacity (Kosar et al., 2007). The antioxidant activity of polyphenols is mainly due to the phenolic hydroxyl group of polyphenols that can stabilize free radicals and inhibit the oxidation of lipids, proteins, and DNA by donating hydrogen and thus reduce the damaging effects of oxidation (Rice‐Evans et al., 1997). Researchers have reported that the TPC has a significant effect on antioxidant activity due to their high reduction ability and their ability to deliver hydrogen to free radicals (Sharifi & Poorakbar, 2015). The results showed that the free radical scavenging activity of encapsulated ITLE ranged from 62.25% to 75.06%, which indicates the high inhibitory activity of Vasmeh extract powder (Table 2). The results also showed that with increasing the carrier concentration, the free radical scavenging power increased. Similar to the results observed in the present study, Malekizadeh et al. and Hesarinejad et al. also reported an increase in the power of free radical scavenging by increasing the concentration of maltodextrin in *Portulaca oleracea* and sumac extract encapsules, respectively (Hesarinejad, Abdollahi Moghaddam, et al., 2021; Malekizadeh et al., 2018). They stated that by increasing the concentration of maltodextrin due to its protective effect, it has caused more protection of compounds with antioxidant activity and as a result, the antioxidant activity of the material has increased.

### 3.2 Ice‐cream characterization

#### 3.2.1 Physical properties of ice creams

Food color is one of the effective quality parameters in product acceptance by the consumer and can indicate food defects (Brosnan & Sun, 2002). The results of this study showed that the lightness of the samples decreased slightly with increasing the concentration of ITLE (*p* > .05) (Table 3). Mahjuri (2012) and Mohammadi et al. (2016) also reported similar results. They stated that the L* parameter decreased by increasing the concentration of *Spirulina platensis* in ice cream and yoghurt, respectively (Mahjuri, 2012; Mohammadi et al., 2016). The values of the a* value are in the range of −120 to +120. Negative and positive values represent green and red, respectively (Varela et al., 2014). The results of this study indicated that with increasing the ITLE concentration, the value of a* increased. The b* values ranged from −120 to +120. Negative values indicate blue, and positive values represent yellow (Varela et al., 2014). Based on the results, with increasing ITLE, a significant decrease was observed in the b* value of ice‐cream samples. In fact, with increasing ITLE in ice cream, the intensity of blue color increased, and the yellowness of the samples decreased.

**TABLE 3:** Physical properties of ice‐cream samples containing encapsulated *Indigofera tinctoria* L. extract.

Ice‐cream samples	Melting rate (g/min)	Overrun (%)	Hardness (g)	Color parameters		
				L*	a*	b*
Control	0.35 ± 0.011^a^	48.26 ± 2.31^b^	3055 ± 98^a^	83.58 ± 2.02^a^	−0.70 ± 0.04^d^	8.38 ± 0.08^a^
0.6ITLE	0.33 ± 0.014^ab^	50.93 ± 1.40^ab^	2943 ± 154^a^	82.86 ± 1.54^a^	−0.23 ± 0.09^c^	7.67 ± 0.11^b^
1.2ITLE	0.31 ± 0.017^bc^	52.74 ± 1.04^a^	2809 ± 187^a^	79.63 ± 2.14^a^	0.52 ± 0.11^b^	6.57 ± 0.14^c^
1.8ITLE	0.29 ± 0.013^c^	53.50 ± 1.53^a^	2769 ± 105^a^	79.33 ± 3.08^a^	0.81 ± 0.09^a^	5.92 ± 0.10^d^

^a–d^ Means of two replicates in the same row with same superscripts do not differ significantly (*p* > .05).

One of the important and influential physical parameters on the quality of ice cream is its melting rate. If the melting rate is too low or too high, it is a defect for ice cream (Khosrow Shahi, Hesarinejad, et al., 2021). When ice cream melts, heat from the ice surface penetrates deep into the ice cream and water molecules flow (Soukoulis et al., 2008). Increasing the viscosity reduces the mobility of water molecules, and these molecules can hardly pass through the ice‐cream mixture molecules (Karaca et al., 2009). The results showed that adding ITLE to the ice cream reduced the melting rate of the samples compared with the control sample (Table 3). Proteins are able to interact with polyphenols and affect the properties of milk products (Özdal et al., 2018). Many studies have also shown that proteins can bind to polyphenols (Arts et al., 2002; Frazier et al., 2010; Hasni et al., 2011; Nagy et al., 2012; Shpigelman et al., 2010; von Staszewski et al., 2012; Yuksel et al., 2010). By binding polyphenols to proteins, they may potentially affect the availability of specific amino acids, as well as change protein structure, which can affect performance (Jakobek, 2015). Polyphenols can act as a bridge between proteins and form a large network. The result of the formation of this protein–polyphenolic network is the production of a gel that traps various compounds within itself. Adding polyphenols to the ice‐cream formulation also forms a gel made of proteins and polyphenols, and this gel is strong enough that it can hold air bubbles, fat cells, and ice crystals inside even after being exposed to high temperatures. In this case, even if the entire ice part of the sample is melted, the gel structure is able to maintain the product structure and its properties (Yildirim‐Elikoglu & Erdem, 2018). Therefore, this reduction in melting rate with the addition of ITLE may be due to the presence of polyphenolic compounds. Erkaya et al. also observed that melting rate is reduced by adding Cape gooseberry to ice‐cream samples. They explained the reason for the decrease in this factor is the presence of compounds in gooseberry that have the property of absorbing water and increasing viscosity (Erkaya et al., 2012).

Overrun is one of the parameters affecting the quality of ice cream and means that the volume of ice cream compared with the initial mixture is affected by the entry of air. Some factors such as the fat content, the total solid content, sweeteners, stabilizers, and processing affect the increase in ice‐cream volume. The amount of air entering the ice cream is important due to the increase in efficiency and profitability, as well as achieving a more desirable texture that is acceptable to the consumer (Khosrow Shahi, Didar, et al., 2021). The results showed that the overrun value in ice‐cream samples increased significantly with increasing the concentration of ITLE (*p* < .05) (Table 3). The highest overrun value was observed in the sample containing the highest ITLE concentration. The reason for this observation can probably be related to the components in this plant extract. It should be noted that excessive overrun causes a foamy state in ice cream, and low overrun prevents a creamy state and causes undesirable organoleptic properties. The overrun value is important in achieving legal standards and increasing economic profits. The values of overrun in this study ranged from 48.26% to 53.50%, which with the addition of ITLE to ice cream, overrun increased by 5.53%, 9.28%, and 10.85% than the control sample.

The resistance of the ice cream to deformation when an external force was applied was used to determine its hardness (Muse & Hartel, 2004). Hardness is a measure of the composition of the ice‐cream mix and its processing conditions (Goff et al., 1995). Hardness is important because it has a direct effect on spoonability. The components of the ice cream (protein, fat, etc.), freezing point, overrun, total solids, and the type of stabilizer used are all factors that influence hardness (Goff et al., 1995). The results showed that with increasing the ITLE in ice cream, the hardness decreased (*p* > .05) (Table 3). According to the results (Table 3), adding 0.6% ITLE to the formulation and increasing it to 1.8% reduced the hardness of the ice creams from 3055 to 2769 g. Some researchers have also reported an inverse correlation between hardness and overrun, which is consistent with our findings. According to research, the higher the hardness, the lower the overrun. This is due to the existence of less air in the ice cream's continuous matrix, which makes it more resistant to the probe of the texture analyzer penetrating it and diminishes the product's spoonability (Goff & Hartel, 2013).

#### 3.2.2 Rheological properties of ice cream mixes

**FIGURE 1:**
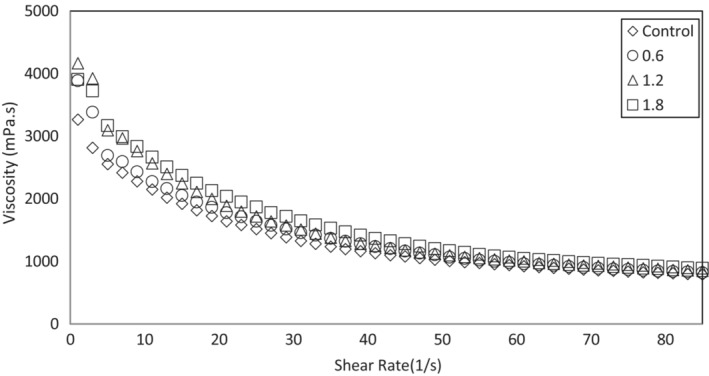
Apparent viscosity at 25°C on shear rate of ice‐cream mix formulation contains different concentrations of encapsulated *Indigofera tinctoria* L. extract.

Viscosity is one of the most important parameters to achieve a suitable formulation, select the type of pump for proper transfer, and design of equipment. The viscosity of the ice‐cream mixture affects the texture and quality of the final product, overrun, creaminess, and the rate of mass and heat transfer (Ruger et al., 2002). The main reason for the increase in viscosity is the type of constituents of the product, their molecular weight, the hydrophilic ability to bond with water, and the formation of a 3D network (Goff, 2002). It has been stated that the effect of protein on increasing viscosity is more important than other compounds (Shama & Sherman, 1966). Although it is impossible to determine with confidence how much viscosity is appropriate for ice‐cream mix, experience indicated that increasing viscosity to a certain amount improves melting resistance, textural properties, organoleptic properties, and product quality. The flow behavior diagram of the ice‐cream mix is shown in Figure 1. These rheological data were fitted with the Herschel–Bulkley model. The high *R* ^2^ and low RMSE obtained by fitting the experimental data to the Herschel–Bulkley model confirmed the model's proper fitting, as shown in Table 4.

**TABLE 4:** Rheological properties of ice‐cream samples

Ice‐cream samples	Herschel–Bulkley model				
	*k* (Pa s^n^)	*n*	*τ*_0_ (Pa)	*R* ^2^	RMSE
Control	11.8 ± 0.2^c^	0.41 ± 0.01^a^	0.8 ± 0.1^d^	0.99	1.04
0.6ITLE	13.7 ± 0.3^b^	0.35 ± 0.01^b^	1.2 ± 0.1^c^	0.99	1.07
1.2ITLE	16.9 ± 0.2^a^	0.33 ± 0.01^b^	1.6 ± 0.1^b^	0.99	1.34
1.8ITLE	17.0 ± 0.3^a^	0.28 ± 0.01^c^	2.4 ± 0.1^a^	0.99	1.33

^a–d^ Means of two replicates in the same row with same superscripts do not differ significantly (*p* > .05).

The flow behavior index values (0.28–0.41) suggested that all of the samples were non‐Newtonian/shear thinning, which was consistent with prior reports (Aime et al., 2001; Akalın et al., 2008; Javidi et al., 2016; Javidi & Razavi, 2018; Karaca et al., 2009; Khosrow Shahi, Didar, et al., 2021; Khosrow Shahi, Hesarinejad, et al., 2021). This behavior results in a more pleasing texture and mouthfeel, as well as more efficient pumping (Bahramparvar et al., 2010). The *n* values of the mixes decreased with a rise in the ITLE content, probably due to the formation of the protein–polyphenolic networks, which are sensitive to shear rate. The results showed that increasing the ITLE concentration increased the consistency coefficient (*k*) of the ice‐cream mixture (Table 4). It was the lowest for the control (11.8 Pa s^n^) and the highest for the mix containing 1.8% ITLE (17.0 Pa s^n^). Probably, one of the reasons for the increase in the consistency coefficient of the ice‐cream mix was the increase in dry matter due to the addition of encapsulated ITLE to ice‐ceam mix. Mohammeed et al. stated that the increase in total solids is one of the factors affecting the increase in consistency coefficient (Mohameed et al., 2004). Table 4 illustrates the yield stress values, which are known to be related to the body, texture, and scoopability of ice cream (Khosrow Shahi, Didar, et al., 2021). 1.8% ITLE caused the most yield stress among the various ITLE concentrations employed in this investigation, which could be owing to the increased intermolecular interactions (Regand & Goff, 2006).

#### 3.2.3 Sensory attributes of ice cream

Sensory evaluation of the samples was performed in terms of color, coldness, coarseness, hardness, and overall acceptance. The results showed that in all parameters, the control ice‐cream sample had a higher acceptance (Table 5). In the color parameter, ice cream containing 1.2% ITLE obtained the highest score following the control sample. There is a relationship between ice cream color and its acceptance by the consumer (Karaman et al., 2014). In the evaluation of the color parameter, different results were observed, which showed a difference between the tastes of the evaluators. A small number of evaluators gave high scores to ice creams containing ITLE and a higher number gave high scores to control ice creams. This was an interesting observation that caused the standard deviation of the color attribute data for the two control samples and 1.8ITLE to be high. The results showed that the two parameters of coldness and coarseness due to the addition of ITLE did not improve significantly (*p* > .05). As can be seen, hardness scores decreased with the addition of ITLE (Table 5), possibly due to an increase in overrun. Staffolo et al. stated that the score of panelists increases as the force required to compress the product increases (Staffolo et al., 2004).

**TABLE 5:** Sensory characteristics of ice‐cream samples

Ice‐cream samples	Color	Coldness	Coarseness	Hardness	Overall acceptance
Control	8.6 ± 0.9^a^	6.5 ± 0.4^a^	6.9 ± 0.4^a^	6.6 ± 0.3^a^	8.0 ± 0.8^a^
0.6ITLE	8.1 ± 0.2^ab^	6.4 ± 0.2^a^	6.8 ± 0.3^a^	6.2 ± 0.2^ab^	7.8 ± 0.6^ab^
1.2ITLE	8.2 ± 0.4^ab^	6.3 ± 0.2^a^	6.8 ± 0.4^a^	6.4 ± 0.1^ab^	7.4 ± 0.5^ab^
1.8ITLE	7.5 ± 1.0^b^	6.3 ± 0.4^a^	6.6 ± 0.5^a^	5.9 ± 0.4^b^	6.8 ± 0.7^b^

^a,b^ Means of two replicates in the same row with same superscripts do not differ significantly (*p* > .05).

## 4 CONCLUSIONS

In recent years, the tendency to consume functional foods has increased. In addition to high nutritional value, functional products have health‐friendly ingredients for the consumer. Consumption of dairy products is a major part of the diet of people around the world and is considered as an indicator of the development of human societies. Ice cream is one of the most delicious dairy products that can be considered as a substrate for beneficial compounds. The results of this study showed that encapsulation of Vasmeh extract with different concentrations of maltodextrin by freeze‐drying method reduced the MC, a_w_ and solubility, bulk density, and tapped density of powders. A significant increase in sample lightness and a significant decrease in a* and b* parameters were observed with increasing maltodextrin concentration. Also, with increasing the concentration of maltodextrin, the TPC and antioxidant activity of powders increased. In ice‐cream samples, with increasing concentration of ITLE, the lightness of the samples decreased, the a* value increased, and the b* value decreased. Addition of ITLE to the ice‐cream formulation caused a decrease in the melting rate and hardness of the samples, an increase in overrun of ice cream, and the viscosity of the ice cream mix. The results of this study showed that ITLE, with its coloring ability and appropriate antioxidant properties, can be used as a desirable additive in the production of useful products with natural color.

## FUNDING INFORMATION

This research did not receive any specific grant from funding agencies in the public, commercial, or not‐for‐profit sectors.

## ACKNOWLEDGEMENTS

Many thanks go to the Research Institute of Food Science and Technology in Mashhad for facilitating the process to conduct this study and providing laboratory equipment for experiments of this research.

## CONFLICT OF INTEREST

There is no conflict of interest.

## CONSENT FOR PUBLICATION

All authors have read and agreed to the published version of the manuscript. All authors read and approved the final manuscript.

## Data Availability

All data generated or analyzed during this study are included in this published article.

## References

[fsn33228-bib-0001] Abadio, F. D. B. , Domingues, A. M. , Borges, S. V. , & Oliveira, V. M. (2004). Physical properties of powdered pineapple (*Ananas comosus*) juice––Effect of malt dextrin concentration and atomization speed. Journal of Food Engineering, 64(3), 285–287.

[fsn33228-bib-0002] Abdullah, E. C. , & Geldart, D. (1999). The use of bulk density measurements as flowability indicators. Powder Technology, 102(2), 151–165.

[fsn33228-bib-0003] Aime, D. B. , Arntfield, S. D. , Malcolmson, L. J. , & Ryland, D. (2001). Textural analysis of fat reduced vanilla ice cream products. Food Research International, 34(2–3), 237–246.

[fsn33228-bib-0004] Akalın, A. S. , Karagözlü, C. , & Ünal, G. (2008). Rheological properties of reduced‐fat and low‐fat ice cream containing whey protein isolate and inulin. European Food Research and Technology, 227(3), 889–895.

[fsn33228-bib-0005] Alizadeh Behbahani, B. , Tabatabaei Yazdi, F. , Shahidi, F. , Hesarinejad, M. A. , Mortazavi, S. A. , & Mohebbi, M. (2017). Plantago major seed mucilage: Optimization of extraction and some physicochemical and rheological aspects. Carbohydrate Polymers, 155, 68–77. 10.1016/j.carbpol.2016.08.051 27702546

[fsn33228-bib-0006] Amiryousefi, M. R. , & Hesarinejad, M. A. (2021). Modeling of kinetic changes of ostrich meat color during deep fat frying by image processing. Journal of Food Science and Technology (Iran), 18(111), 361–369.

[fsn33228-bib-0007] Anusuya, N. , & Manian, S. (2013). Antioxidant and free radical scavenging potential of different solvent extracts of *Indigofera tinctoria* L. leaves. International Journal of Pharmacy and Pharmaceutical Sciences, 5(1), 142–147.

[fsn33228-bib-0009] AOAC . (1990). In K. Helrich (Ed.), Official methods of analysis (15th ed.). Association of Official Analytical Chemists Inc.

[fsn33228-bib-0010] Arici, M. , Karasu, S. , Baslar, M. , Toker, O. S. , Sagdic, O. , & Karaagacli, M. (2016). Tulip petal as a novel natural food colorant source: Extraction optimization and stability studies. Industrial Crops and Products, 91, 215–222.

[fsn33228-bib-0011] Arts, M. J. T. J. , Haenen, G. R. M. M. , Wilms, L. C. , Beetstra, S. A. J. N. , Heijnen, C. G. M. , Voss, H.‐P. , & Bast, A. (2002). Interactions between flavonoids and proteins: Effect on the total antioxidant capacity. Journal of Agricultural and Food Chemistry, 50(5), 1184–1187. 11853501 10.1021/jf010855a

[fsn33228-bib-0012] Azeredo, H. M. C. (2009). Betalains: Properties, sources, applications, and stability—A review. International Journal of Food Science & Technology, 44(12), 2365–2376.

[fsn33228-bib-0013] Bahramparvar, M. , Razavi, S. M. A. , & Khodaparast, M. H. H. (2010). Rheological characterization and sensory evaluation of a typical soft ice cream made with selected food hydrocolloids. Food Science and Technology International, 16(1), 79–88. 10.1177/1082013209353244 21339124

[fsn33228-bib-0014] Bayat Tork, M. , Vazifedoost, M. , Hesarinejad, M. A. , Didar, Z. , & Shafafi Zenoozian, M. (2022). Predictive modeling to determine the shelf life of snacks enriched with dragees containing *Spirulina platensis* . Journal of Food Science and Technology (Iran), 18(121), 335–345.

[fsn33228-bib-0015] Brosnan, T. , & Sun, D.‐W. (2002). Inspection and grading of agricultural and food products by computer vision systems—A review. Computers and Electronics in Agriculture, 36(2–3), 193–213.

[fsn33228-bib-0016] Cano‐Chauca, M. , Stringheta, P. C. , Ramos, A. M. , & Cal‐Vidal, J. (2005). Effect of the carriers on the microstructure of mango powder obtained by spray drying and its functional characterization. Innovative Food Science & Emerging Technologies, 6(4), 420–428.

[fsn33228-bib-0017] Caparino, O. A. , Tang, J. , Nindo, C. I. , Sablani, S. S. , Powers, J. R. , & Fellman, J. K. (2012). Effect of drying methods on the physical properties and microstructures of mango (Philippine ‘Carabao’ var.) powder. Journal of Food Engineering, 111(1), 135–148.

[fsn33228-bib-0018] Chou, P. , Matsui, S. , Misaki, K. , & Matsuda, T. (2007). Isolation and identification of xenobiotic aryl hydrocarbon receptor ligands in dyeing wastewater. Environmental Science & Technology, 41(2), 652–657. 17310736 10.1021/es061500g

[fsn33228-bib-0019] Chranioti, C. , Nikoloudaki, A. , & Tzia, C. (2015). Saffron and beetroot extracts encapsulated in maltodextrin, gum Arabic, modified starch and chitosan: Incorporation in a chewing gum system. Carbohydrate Polymers, 127, 252–263. 25965482 10.1016/j.carbpol.2015.03.049

[fsn33228-bib-0020] de Vos, P. , Faas, M. M. , Spasojevic, M. , & Sikkema, J. (2010). Encapsulation for preservation of functionality and targeted delivery of bioactive food components. International Dairy Journal, 20(4), 292–302.

[fsn33228-bib-0021] Staffolo, M. D. , Bertola, N. , & Martino, M. (2004). Influence of dietary fiber addition on sensory and rheological properties of yogurt. International Dairy Journal, 14(3), 263–268.

[fsn33228-bib-0022] Desai, K. G. H. , & Jin Park, H. (2005). Recent developments in microencapsulation of food ingredients. Drying Technology, 23(7), 1361–1394.

[fsn33228-bib-0023] Durmaz, Y. , Kilicli, M. , Toker, O. S. , Konar, N. , Palabiyik, I. , & Tamtürk, F. (2020). Using spray‐dried microalgae in ice cream formulation as a natural colorant: Effect on physicochemical and functional properties. Algal Research, 47, 101811.

[fsn33228-bib-0024] Embuscado, M. E. (2015). Spices and herbs: Natural sources of antioxidants—A mini review. Journal of Functional Foods, 18, 811–819.

[fsn33228-bib-0025] Erkaya, T. , Dağdemir, E. , & Şengül, M. (2012). Influence of cape gooseberry (*Physalis peruviana* L.) addition on the chemical and sensory characteristics and mineral concentrations of ice cream. Food Research International, 45(1), 331–335.

[fsn33228-bib-0026] Fang, Z. , & Bhandari, B. (2010). Encapsulation of polyphenols—A review. Trends in Food Science & Technology, 21(10), 510–523.

[fsn33228-bib-0027] Fazaeli, M. , Emam‐Djomeh, Z. , Ashtari, A. K. , & Omid, M. (2012). Effect of spray drying conditions and feed composition on the physical properties of black mulberry juice powder. Food and Bioproducts Processing, 90(4), 667–675.

[fsn33228-bib-0028] Fennema, O. R. (Ed.) (1996). Water and ice. In Food chemistry (3rd ed., pp. 42–49). Marcel Dekker Inc.

[fsn33228-bib-0029] Frazier, R. A. , Deaville, E. R. , Green, R. J. , Stringano, E. , Willoughby, I. , Plant, J. , & Mueller‐Harvey, I. (2010). Interactions of tea tannins and condensed tannins with proteins. Journal of Pharmaceutical and Biomedical Analysis, 51(2), 490–495. 19553056 10.1016/j.jpba.2009.05.035

[fsn33228-bib-0030] Genc Polat, D. , Durmaz, Y. , Konar, N. , Toker, O. S. , Palabiyik, I. , & Tasan, M. (2020). Using encapsulated *Nannochloropsis oculata* in white chocolate as coloring agent. Journal of Applied Phycology, 32(5), 3077–3088.

[fsn33228-bib-0031] Ghaderi, S. , Mazaheri Tehrani, M. , & Hesarinejad, M. A. (2021). Qualitative analysis of the structural, thermal and rheological properties of a plant ice cream based on soy and sesame milks. Food Science & Nutrition, 9(3), 1289–1298. 33747445 10.1002/fsn3.2037 PMC7958547

[fsn33228-bib-0032] Goff, H. D. (2002). Formation and stabilisation of structure in ice‐cream and related products. Current Opinion in Colloid & Interface Science, 7(5–6), 432–437.

[fsn33228-bib-0033] Goff, H. D. , & Hartel, R. W. (2013). Ice cream. Springer Science & Business Media.

[fsn33228-bib-0034] Goff, H. D. , Freslon, B. , Sahagian, M. E. , Hauber, T. D. , Stone, A. P. , & Stanley, D. W. (1995). Structural development in ice cream—Dynamic rheological measurements. Journal of Texture Studies, 26(5), 517–536.

[fsn33228-bib-0035] Gong, Z. , Zhang, M. , Mujumdar, A. S. , & Sun, J. (2007). Spray drying and agglomeration of instant bayberry powder. Drying Technology, 26(1), 116–121.

[fsn33228-bib-0036] Goula, A. M. , & Adamopoulos, K. G. (2010). A new technique for spray drying orange juice concentrate. Innovative Food Science & Emerging Technologies, 11(2), 342–351.

[fsn33228-bib-0037] Goula, A. M. , Adamopoulos, K. G. , & Kazakis, N. A. (2004). Influence of spray drying conditions on tomato powder properties. Drying Technology, 22(5), 1129–1151.

[fsn33228-bib-0038] Grabowski, J. A. , Truong, V. , & Daubert, C. R. (2006). Spray‐drying of amylase hydrolyzed sweetpotato puree and physicochemical properties of powder. Journal of Food Science, 71(5), E209–E217.

[fsn33228-bib-0039] Grabowski, J. A. , Truong, V.‐D. , & Daubert, C. R. (2008). Nutritional and rheological characterization of spray dried sweetpotato powder. LWT‐ Food Science and Technology, 41(2), 206–216.

[fsn33228-bib-0040] Han, R. (1994). Highlight on the studies of anticancer drugs derived from plants in China. Stem Cells, 12(1), 53–63. 8142920 10.1002/stem.5530120110

[fsn33228-bib-0041] Hasni, I. , Bourassa, P. , Hamdani, S. , Samson, G. , Carpentier, R. , & Tajmir‐Riahi, H.‐A. (2011). Interaction of milk α‐and β‐caseins with tea polyphenols. Food Chemistry, 126(2), 630–639.

[fsn33228-bib-0042] Hesarinejad, M. A. , Abdollahi Moghaddam, M. R. , Jafarzadeh, M. , & Rezaee Oghazi, M. (2021). The study of physicochemical and antioxidant properties of encapsulated Portulaca oleracea aqueous extract prepared by spray drying method. Innovative Food Technologies, 8(3), 325–335.

[fsn33228-bib-0043] Hesarinejad, M. A. , Lorenzo, J. M. , & Rafe, A. (2021). Influence of gelatin/guar gum mixture on the rheological and textural properties of restructured ricotta cheese. Carbohydrate Polymer Technologies and Applications, 2, 100162.

[fsn33228-bib-0044] Horuz, E. , Altan, A. , & Maskan, M. (2012). Spray drying and process optimization of unclarified pomegranate (*Punica granatum*) juice. Drying Technology, 30(7), 787–798.

[fsn33228-bib-0045] Jakobek, L. (2015). Interactions of polyphenols with carbohydrates, lipids and proteins. Food Chemistry, 175, 556–567. 25577120 10.1016/j.foodchem.2014.12.013

[fsn33228-bib-0046] Jangam, S. V. , & Thorat, B. N. (2010). Optimization of spray drying of ginger extract. Drying Technology, 28(12), 1426–1434.

[fsn33228-bib-0047] Javidi, F. , & Razavi, S. (2018). Rheological, physical and sensory characteristics of light ice cream as affected by selected fat replacers. Journal of Food Measurement and Characterization, 12(3), 1872–1884.

[fsn33228-bib-0048] Javidi, F. , Razavi, S. M. A. , Behrouzian, F. , & Alghooneh, A. (2016). The influence of basil seed gum, guar gum and their blend on the rheological, physical and sensory properties of low fat ice cream. Food Hydrocolloids, 52, 625–633.

[fsn33228-bib-0049] Karaca, O. B. , Güven, M. , Yasar, K. , Kaya, S. , & Kahyaoglu, T. (2009). The functional, rheological and sensory characteristics of ice creams with various fat replacers. International Journal of Dairy Technology, 62(1), 93–99.

[fsn33228-bib-0050] Karaman, S. , Toker, Ö. S. , Yüksel, F. , Çam, M. , Kayacier, A. , & Dogan, M. (2014). Physicochemical, bioactive, and sensory properties of persimmon‐based ice cream: Technique for order preference by similarity to ideal solution to determine optimum concentration. Journal of Dairy Science, 97(1), 97–110. 24268400 10.3168/jds.2013-7111

[fsn33228-bib-0051] Khosrow Shahi, S. , Didar, Z. , Hesarinejad, M. A. , & Vazifedoost, M. (2021). Optimized pulsed electric field‐assisted extraction of biosurfactants from Chubak (*Acanthophyllum squarrosum*) root and application in ice cream. Journal of the Science of Food and Agriculture, 101(9), 3693–3706. 33301192 10.1002/jsfa.11000

[fsn33228-bib-0052] Khosrow Shahi, S. , Hesarinejad, M. A. , Didar, Z. , & Vazifedoost, M. (2021). Investigation of the effect of using Chubak extract on physicochemical and sensory properties of ice milk. Food Science and Technology, 18(114), 225–235.

[fsn33228-bib-0053] Koocheki, A. , Hesarinejad, M. A. , & Mozafari, M. R. (2022). Lepidium perfoliatum seed gum: Investigation of monosaccharide composition, antioxidant activity and rheological behavior in presence of salts. Chemical and Biological Technologies in Agriculture, 9(1), 1–14.

[fsn33228-bib-0054] Kosar, M. , Bozan, B. , Temelli, F. , & Baser, K. H. C. (2007). Antioxidant activity and phenolic composition of sumac (*Rhus coriaria* L.) extracts. Food Chemistry, 103(3), 952–959.

[fsn33228-bib-0055] Mahjuri, N. (2012). Investigation of physical and sensory properties of ice cream enriched with Spirulina platensis. University of Tehran.

[fsn33228-bib-0056] Malekizadeh, N. , Peighambardoust, S. H. , Oladghaffari, A. , & Sarabandi, K. (2018). Effects of different concentrations of maltodextrin and drying temperatures of spray drying process on physicochemical properties of encapsulated sumac extract. Iranian Food Science and Technology Research Journal, 14(2), 321–334.

[fsn33228-bib-0057] Mishra, P. , Mishra, S. , & Mahanta, C. L. (2014). Effect of maltodextrin concentration and inlet temperature during spray drying on physicochemical and antioxidant properties of amla (*Emblica officinalis*) juice powder. Food and Bioproducts Processing, 92(3), 252–258.

[fsn33228-bib-0058] Mizutani, T. (2009). Toxicity of xanthene food dyes by inhibition of human drug‐metabolizing enzymes in a noncompetitive manner. Journal of Environmental and Public Health, 2009, 1–9. 10.1155/2009/953952 PMC2778353 20041016

[fsn33228-bib-0059] Mohameed, H. A. , Abu‐Jdayil, B. , & Al‐Shawabkeh, A. (2004). Effect of solids concentration on the rheology of labneh (concentrated yogurt) produced from sheep milk. Journal of Food Engineering, 61(3), 347–352.

[fsn33228-bib-0060] Mohammadi, F. , Fadaee, V. , & Khosravi, K. (2016). Influence of different concentrations of *Spirulina platensis* on some physicochemical and sensory properties of probiotic spinach yoghurt. Journal of Food Research, 26(2), 127–143.

[fsn33228-bib-0061] Mosén, K. , Bäckström, K. , Thalberg, K. , Schaefer, T. , Kristensen, H. G. , & Axelsson, A. (2005). Particle formation and capture during spray drying of inhalable particles. Pharmaceutical Development and Technology, 9(4), 409–417. 10.1081/pdt-200035795 15581077

[fsn33228-bib-0062] Muse, M. R. , & Hartel, R. W. (2004). Ice cream structural elements that affect melting rate and hardness. Journal of Dairy Science, 87(1), 1–10. 14765804 10.3168/jds.S0022-0302(04)73135-5

[fsn33228-bib-0063] Nadeem, H. Ş. , Torun, M. , & Özdemir, F. (2011). Spray drying of the mountain tea (*Sideritis stricta*) water extract by using different hydrocolloid carriers. LWT‐ Food Science and Technology, 44(7), 1626–1635.

[fsn33228-bib-0064] Nagy, K. , Courtet‐Compondu, M.‐C. , Williamson, G. , Rezzi, S. , Kussmann, M. , & Rytz, A. (2012). Non‐covalent binding of proteins to polyphenols correlates with their amino acid sequence. Food Chemistry, 132(3), 1333–1339. 29243619 10.1016/j.foodchem.2011.11.113

[fsn33228-bib-0065] Naji‐Tabasi, S. , Emadzadeh, B. , Shahidi‐Noghabi, M. , Abbaspour, M. , & Akbari, E. (2021). Physico‐chemical and antioxidant properties of barberry juice powder and its effervescent tablets. Chemical and Biological Technologies in Agriculture, 8(1), 1–11.

[fsn33228-bib-0066] Narender, T. , Khaliq, T. , & Puri, A. (2006). Antidyslipidemic activity of Furano‐flavonoids isolated from Indigofera tinctoria. Bioorganic & Medicinal Chemistry Letters, 16(13), 3411–3414. 16644212 10.1016/j.bmcl.2006.04.001

[fsn33228-bib-0067] Nikjoo, R. , Peighambardoust, S. H. , & Olad Ghaffari, A. (2019). Effect of spray drying on physicochemical characteristics and quality of peppermint powder. Food Science and Technology, 16(95), 99–109.

[fsn33228-bib-0068] Özdal, T. , Yalçınkaya, İ. E. , Toydemir, G. , & Çapanoglu, E. (2018). Polyphenol–protein interactions and changes in functional properties and digestibility .

[fsn33228-bib-0069] Öztürk, H. İ. , Demirci, T. , & Akın, N. (2018). Production of functional probiotic ice creams with white and dark blue fruits of *Myrtus communis*: The comparison of the prebiotic potentials on lactobacillus casei 431 and functional characteristics. LWT, 90, 339–345.

[fsn33228-bib-0070] Quek, S. Y. , Chok, N. K. , & Swedlund, P. (2007). The physicochemical properties of spray‐dried watermelon powders. Chemical Engineering and Processing: Process Intensification, 46(5), 386–392.

[fsn33228-bib-0071] Rad, M. A. , Emam‐Djomeh, Z. , & Asadi, H. (2016). Effect of spray drying conditions on the physicochemical properties of cornelian pherry juice powder. Journal of Food Science & Technology (2008‐8787), 12(50), 62–73.

[fsn33228-bib-0072] Regand, A. , & Goff, H. D. (2006). Ice recrystallization inhibition in ice cream as affected by ice structuring proteins from winter wheat grass. Journal of Dairy Science, 89(1), 49–57. 16357267 10.3168/jds.S0022-0302(06)72068-9

[fsn33228-bib-0073] Rezaei, N. , Salimi, A. , Shemshadi, G. , Kazemzadeh, M. , & Jebeli Javan, A. (2019). Optimization of extraction conditions of antioxidant and polyphenolic compounds of Ferula Persica extract by using response surface methodology. Food Science and Technology, 15(85), 151–164.

[fsn33228-bib-0074] Rezagholi, F. , & Hesarinejad, M. A. (2017). Integration of fuzzy logic and computer vision in intelligent quality control of celiac‐friendly products. Procedia Computer Science, 120, 325–332. 10.1016/j.procs.2017.11.246

[fsn33228-bib-0075] Rezaiyan Attar, F. , Sedaghat, N. , Yeganehzad, S. , Pasban, A. , & Hesarinejad, M. A. (2021). Shelf life modeling of Badami's fresh pistachios coated with chitosan under modified atmosphere packaging conditions. Food Science and Technology, 18(114), 181–194.

[fsn33228-bib-0076] Rice‐Evans, C. , Miller, N. , & Paganga, G. (1997). Antioxidant properties of phenolic compounds. Trends in Plant Science, 2(4), 152–159.

[fsn33228-bib-0077] Ruger, P. R. , Baer, R. J. , & Kasperson, K. M. (2002). Effect of double homogenization and whey protein concentrate on the texture of ice cream. Journal of Dairy Science, 85(7), 1684–1692. 12201518 10.3168/jds.S0022-0302(02)74241-0

[fsn33228-bib-0078] Saini, R. , Garg, V. , & Dangwal, K. (2013). Effect of extraction solvents on polyphenolic composition and antioxidant, antiproliferative activities of Himalyan bayberry (*Myrica esculenta*). Food Science and Biotechnology, 22(4), 887–894.

[fsn33228-bib-0079] Salehi, M. , Khajehrahimi, A. , & Hesarinejad, M. A. (2021). The effect of *Dunaliella salina* on physicochemical and sensory properties of yogurt. Food Science and Technology, 18(117), 95–107.

[fsn33228-bib-0080] Santhalakshmy, S. , Bosco, S. J. D. , Francis, S. , & Sabeena, M. (2015). Effect of inlet temperature on physicochemical properties of spray‐dried jamun fruit juice powder. Powder Technology, 274, 37–43.

[fsn33228-bib-0081] Sarabandi, K. , & Peighambardoust, S. H. (2015). Effect of some production parameters and storage time on the flowability characteristics of spray‐dried malt extract powder. Iranian Journal of Nutrition Sciences & Food Technology, 10(1), 51–60.

[fsn33228-bib-0082] Shahidi, F. , Varidi, M. , Mohebbi, M. , Noshad, M. , & Noshad, M. (2014). Optimization of spray drying of pomegranate juice using response surface methodology. Research and Innovation in Food Science and Technology, 3(2), 129–142. 10.22101/jrifst.2014.08.23.323

[fsn33228-bib-0083] Shama, F. , & Sherman, P. (1966). The texture of ice cream 2. Rheological properties of frozen ice cream. Journal of Food Science, 31(5), 699–706.

[fsn33228-bib-0084] Sharifi, F. , & Poorakbar, L. (2015). The survey of antioxidant properties of phenolic compounds in fresh and dry hybrid barberry fruits (*Berberis integerrima* × *vulgaris*). Cumhuriyet Üniversitesi Fen Edebiyat Fakültesi Fen Bilimleri Dergisi, 36(3), 1609–1617.

[fsn33228-bib-0085] Shpigelman, A. , Israeli, G. , & Livney, Y. D. (2010). Thermally‐induced protein–polyphenol co‐assemblies: Beta lactoglobulin‐based nanocomplexes as protective nanovehicles for EGCG. Food Hydrocolloids, 24(8), 735–743.

[fsn33228-bib-0086] Soukoulis, C. , Chandrinos, I. , & Tzia, C. (2008). Study of the functionality of selected hydrocolloids and their blends with κ‐carrageenan on storage quality of vanilla ice cream. LWT‐ Food Science and Technology, 41(10), 1816–1827.

[fsn33228-bib-0087] Stone, H. , & Sidel, J. L. (Eds.) (2004). Introduction to sensory evaluation. In Sensory evaluation practices (3rd ed., pp. 1–19). Academic Press.

[fsn33228-bib-0088] Tewa‐Tagne, P. , Briançon, S. , & Fessi, H. (2007). Preparation of redispersible dry nanocapsules by means of spray‐drying: Development and characterisation. European Journal of Pharmaceutical Sciences, 30(2), 124–135. 17150339 10.1016/j.ejps.2006.10.006

[fsn33228-bib-0089] Tonon, R. V. , Brabet, C. , & Hubinger, M. D. (2010). Anthocyanin stability and antioxidant activity of spray‐dried açai (*Euterpe oleracea* Mart.) juice produced with different carrier agents. Food Research International, 43(3), 907–914.

[fsn33228-bib-0090] Trgo, C. , Koxholt, M. , & Kessler, H. G. (1999). Effect of freezing point and texture regulating parameters on the initial ice crystal growth in ice cream. Journal of dairy science, 82(3), 460–465.

[fsn33228-bib-0091] Tuyen, C. K. , Nguyen, M. H. , & Roach, P. D. (2010). Effects of spray drying conditions on the physicochemical and antioxidant properties of the Gac (*Momordica cochinchinensis*) fruit aril powder. Journal of Food Engineering, 98(3), 385–392.

[fsn33228-bib-0092] Van Kleef, E. , Van Trijp, H. C. M. , Luning, P. , & Jongen, W. M. F. (2002). Consumer‐oriented functional food development: How well do functional disciplines reflect the ‘voice of the consumer’? Trends in Food Science & Technology, 13(3), 93–101.

[fsn33228-bib-0093] Varela, P. , Pintor, A. , & Fiszman, S. (2014). How hydrocolloids affect the temporal oral perception of ice cream. Food Hydrocolloids, 36, 220–228.

[fsn33228-bib-0094] von Staszewski, M. , Jara, F. L. , Ruiz, A. L. T. G. , Jagus, R. J. , Carvalho, J. E. , & Pilosof, A. M. R. (2012). Nanocomplex formation between β‐lactoglobulin or caseinomacropeptide and green tea polyphenols: Impact on protein gelation and polyphenols antiproliferative activity. Journal of Functional Foods, 4(4), 800–809.

[fsn33228-bib-0095] Yildirim‐Elikoglu, S. , & Erdem, Y. K. (2018). Interactions between milk proteins and polyphenols: Binding mechanisms, related changes, and the future trends in the dairy industry. Food Reviews International, 34(7), 665–697.

[fsn33228-bib-0096] Yuksel, Z. , Avci, E. , & Erdem, Y. K. (2010). Characterization of binding interactions between green tea flavanoids and milk proteins. Food Chemistry, 121(2), 450–456.

[fsn33228-bib-0097] Zendeboodi, F. , Yeganehzad, S. , & Sadeghian, A. R. (2018). Production of carbohydrate‐protein based soft drink powder containing date syrup by spray dryer: Evaluation effect of drying carriers on physical properties of the powdered drink. Journal of Food Science and Technology, 15(78), 43–54.

[fsn33228-bib-0098] Zuidam, N. J. , & Nedovic, V. (2010). Encapsulation technologies for active food ingredients and food processing . 10.1007/978-1-4419-1008-0

